# Ruta montana L. leaf essential oil and extracts: characterization of bioactive compounds and suppression of crown gall disease

**DOI:** 10.17179/excli2014-655

**Published:** 2015-01-13

**Authors:** Inés Hammami, Slim Smaoui, Anis Ben Hsouna, Naceur Hamdi, Mohamed Ali Triki

**Affiliations:** 1 Unité de Recherche Protection des Plantes Cultivées et Environnement, Institut de l'Olivier, Sfax BP1087, Tunisia; 2 Laboratoire de Microorganismes et de Biomolécules du Centre de Biotechnology de Sfax. Route Sidi Mansour Km 6 B.P. 117, 3018 Sfax- Tunisia; 3 Department of Life Sciences, Faculty of Sciences of Gafsa, Zarroug 2112, Gafsa, Tunisia; 4 College of Science and Arts, Al-Rass, P.O. Box 53, Qassim University, Saudi Arabia; 5 High Institute of Environmental Science and Technologies (HIEST-Borj Cedria, Tunisia)

**Keywords:** Ruta montana, antifungal activity, antibacterial activity, essential oil, in vivo assay

## Abstract

The aims of this study were to assess the antimicrobial efficacy of the leaf essential oil and the leaf extracts of *R. montana *against *Botrytis cinerea*, *Fusarium oxysporum*, *Verticillium dahliae,* *Aspergillus oryzae* and *Fusarium solani.* The oil (1.000 µg/disk) and the extracts (1.500 µg/disk) revealed a remarkable antifungal effect against the tested plant pathogenic fungi with a radial growth inhibition percentage of 40.0-80.0 % and 5.0-58.0 %, respectively along with their respective MIC values ranging from 100 to 1100 µg/mL and 250 to 3000 µg/mL. The oil had a strong detrimental effect on spore germination of all the tested plant pathogens along with the concentration as well as time-dependent kinetic inhibition of *Fusarium oxysporum*. Also, the oil exhibited a potent *in vivo* antifungal effect against *Botrytis cinerea* on tomato plants. Experiments carried out in plant revealed that the essential oil was slightly effective in suppression of gall formation induced by *Agrobacterium tumefaciens* on bitter almond. The results of this study indicate that the oil and extracts of *R. montana *leaves could become natural alternatives to synthetic fungicides to control certain important plant microbial diseases. The GC-MS analysis determined that 28 compounds, which represented 89.03 % of total oil, were present in the oil containing mainly 1-butene, methylcyclopropane, 2-butene and caryophyllene oxide.

## Abbreviations

***CLE***: chloroform leaf extract; ***DMSO***: dimethylsulfoxide; ***ELE***: ethyl acetate leaf extract; ***GC-MS***: Gas chromatography-mass spectrometry; ***HLE***: hexane leaf extract; ***MIC***: Minimum inhibitory concentration; ***MLE***: methanol leaf extract 

## Introduction

Plants are constantly exposed and threatened by a variety of pathogenic microorganisms present in their environments. Diseases caused by plant pathogenic bacteria and fungi significantly contribute to the overall loss in crop yield worldwide (Montesinos, 2007[18]; Savary et al., 2006[23]). In an effort to combat diseases, plants have devised various mechanisms and compounds to fend off microbial invaders. Despite the existence of defense mechanisms, plants are exposed to attack by plant pathogenic microorganisms. Many phytopathogens including *Agrobacterium tumefaciens *(Crown gall), *Pseudomonas savastanoi *pv*. savastanoi* (olive knot disease), *Botrytis cinerea* (grey mould), *Fusarium oxysporum* (vascular wilt), *Verticillium dahlia *(vascular wilt) and *Fusarium solani* (fruit rot) reduce the shelf life and market values of food commodities and render them unfit for human consumption. Widespread use of pesticides has significant drawbacks including cost, handling hazards, pesticide residues, and threats to human health and the environment (Paster and Bullerman, 1988[21]). For many years, a variety of different synthetic chemicals have been extensively used as antifungal and antibacterial agents to inhibit the growth of plant pathogenic fungi and bacteria. However, there is a series of problems against the effective use of these chemicals in areas where bacteria and fungi have developed resistance (Brent and Hollomon, 1998[7]). Public awareness of these factors has increased interest in finding safer alternative protectants to replace synthetic chemical pesticides. Some synthetic pesticides can also cause environmental pollution owing to their slow biodegradation in the environment and even sometimes being carcinogenic toward humans and wild animals (Barnard et al., 1997[5]; Daoubi et al., 2005[10]). Note also that some of the antimycotic compounds, such as members of the azole family, which are widely applied for plant protection, have derivatives that either are used medically or are under clinical evaluation (Walsh et al., 2000[28]). Therefore, it is anticipated that their extensive use in agriculture will increase the risk of selection of resistant phenotypes of human-pathogenic fungi from the surrounding environment or directly from the fungal flora of the human consumer during prolonged exposure to the compounds that are used both in agriculture and in medicine (Barker and Rogers, 2006[4]). 

Research focused on plant-derived fungicides and bactericides and their possible applications in agriculture are being intensified as these are having enormous potential to inspire and influence modern agro-chemical research (Duke, 1990[11]). There has been a growing interest in the research of the possible use of the plant-derived natural fungicides and bactericide such as essential oil and plant extracts, which can be relatively less damaging for pest and disease control in agriculture (Costa et al., 2000[9]; Simlai et al., 2014[24]). Also the plants have long been recognized as providing a potential source of chemical compounds or more commonly, products known as phytochemicals including essential oils and extracts (Negi et al., 2005[20]; Xiao et al., 2014[29]). *Ruta* is a genus of strongly scented evergreen subshrubs 20-60 cm tall, in the family Rutaceae, native to the Mediterranean region, Macaronesia and southwest Asia. There are perhaps 8 to 40 species in the genus. A well-known species is the Common Rue. 

According to *The Oxford Book of Health Foods* (Vaughan and Judd, 2003[27]), extracts from rue have been used to treat eyestrain, sore eyes, and as an insect repellent. Rue has been used internally as an antispasmodic, as a treatment for menstrual problems, as an abortifacient, and as a sedative but there is no evidence of any effect against plant pathogens. Hence, efforts have been given to evaluate the potential role of the essential oil and the extracts from *Ruta montana *L*.* leaves as natural antimicrobial agents. 

The present study was undertaken to investigate the potential use of the leaf essential oil and leaf extracts of *R. montana *L. for the biological control of some important plant phytopathogenic bacteria and fungi causing severe damage to crop, vegetable and ornamental plants. Moreover the chemical composition of the essential oil was examined. 

## Materials and Methods

### Plant materials

Leaves of *R. montana L. *were collected from the local area of Sfax (Tunisia, 35.23° N and 11.11° E), in May 2009. After the botanic identification of the species, a voucher specimen (IO 04) was deposited in the herbarium of the laboratory (Institut de l'Olivier de Sfax) for future reference.

### Isolation of the leaf essential oil

The air-dried leaves of *R. montana *(200 g) were subjected to hydrodistillation for 3 h. The oil, with a yield of 0.26 % (w/w), was dried over anhydrous sodium sulfate Na_2_SO_4_ and, after it was filtered, stored in a sealed vial at 4 °C until tested.

### Preparation of leaf extracts

The air-dried leaves of* R. montana *were pulverized into a powdered form. The dried powder (50 g) was extracted with 70 % hexane, chloroform (CHCl_3_), ethyl acetate (EtOAc) and methanol (MeOH) separately at room temperature and the solvents from the combined extracts were evaporated by vacuum rotary evaporator. The extraction process resulted in hexane (3.8 g), chloroform (4.5 g), ethyl acetate (6.3 g) and methanol (6.1 g) extracts with their respective yields of 7.5, 9.0, 13.6 and 12.2 %.

### Plant pathogenic bacteria and fungi

The fungal species used in the experiment were* B. cinerea, Rhizoctonia solani, F. oxysporum,* *Verticillium dahliae,* *Aspergillus oryzae* and *F. solani*. *V. dahliae* was originally isolated from rotted collar and root of olive trees exhibiting dieback and death in several olive growing areas in Tunisia. *R. solani, F. solani*. were originally isolated from tomato plants exhibiting symptoms of tomato damping off. They were identified by Olive Tree Institute of Sfax and kindly provided by Dr. Mohamed Ali Triki. *B. cinerea, F*. *oxysporum and A. oryzae* were kindly provided from the culture collection of Centre of Biotechnology of Sfax. The isolates were stored at 4 °C in tubes containing potato dextrose agar (PDA), and at -20 °C in tryptone salt medium (tryptone: 1 g, NaCl: 8.5 g, Tween 20: 1 %, glycerol: 15 % and distilled water: 1 l). Bacterial strains* Ag. tumefaciens* C58,* Ag. rhizogene, Ag. vitis *CFBP 2678^T^ and* P. savastanoi *pv* savastanoi* IVIA 1628 (kindly provided from the culture collection of The Olive Tree Institute of Sfax) were grown on Luria Bertani (LB) agar medium plates.

### Preparation of spore suspension and test samples

The fungi were grown on potato dextrose agar (PDA) plates at 25 °C for 2-7 days, after which, spores were harvested from sporulating colonies and suspended in sterile distilled water containing 0.1 % (v/v) Tween 20. The concentrations of spores in suspension were adjusted to 1.0 x 10^8^ spores/mL.

To prepare the stock solutions of essential oil and leaf extracts, the essential oil was dissolved in dimethylsulfoxide (DMSO) separately, whereas the leaf extracts were dissolved in their respective solvents (hexane, chloroform, ethyl acetate and methanol). Samples with known weights were further diluted with 5 % of the respective solvents used to prepare test samples, where the final concentration of the solvent was 0.5 % (v/v). 

### Biological assay of antimicrobial activities 

#### Antifungal activity assay 

Petri dishes (9 cm diameter) containing 20 mL of potato dextrose agar (PDA) were used for the antifungal activity assay, performed on solid media by the disk diffusion method (Duru et al., 2003[12]). Sterile Whatman paper disks of 6 mm diameter were pierced in the agar, equidistant and near the border, where the essential oil (1.000 µg/disk) and the leaf extracts of hexane, chloroform, ethyl acetate and methanol (1.500 µg/disk) were used separately. An agar plug of fungal inoculums (6 mm diameter) was removed from a previous culture of all the fungal strains tested and placed upside down in the centre of the Petri dishes. The plates were incubated at 25 °C for 2-7 days, until the growth in the control plates reached the edge of the plates. The plates without the oil and extracts were used as negative controls. The plates were used in triplicate for each treatment. The relative growth inhibition of the treatment compared to the negative control was calculated as a percentage, using the following formula:

Inhibition (%) = (1- radial growth of treatment (mm)/radial growth of control (mm)) x 100

#### Antibacterial assay

The determination of the antibacterial effect of the oil and extracts was tested according to the agar well diffusion assay (AWDA) (Tagg and McGiven, 1971[26]). The diameters of the inhibition zones were measured. The plates were incubated at 30 °C for 24h. The plates were used in triplicate for each treatment. DMSO, hexane, chloroform, ethyl acetate and methanol were used as control.

Prior to use, the bacterial strains were cultured overnight on nutrient broth (Difco) and inocula were prepared by adjusting the turbidity of each bacterial culture to reach an optical comparison to that of a 0.5 McFarland standard, corresponding to approximately 15 x 10^6^ CFU/ mL. 

#### Antifungal susceptibility assay

The *in vitro* susceptibility of plant pathogenic fungi was determined by the minimum inhibitory concentration determination method (Murray et al., 1995[19]). The minimum inhibitory concentrations (MICs) of the leaf essential oil and the extracts (hexane, chloroform, ethyl acetate and methanol) were determined by two-fold serial dilution against *B. cinerea*, *F. oxysporum*, *V. dahliae,* *A. oryzea* and *F. solani*. The samples of the oil (4 µl) were dissolved in 5 % DMSO, whereas the extracts (8 µl) were dissolved in their respective solvents (hexane, chloroform, ethyl acetate and methanol). These solutions were serially diluted with their respective 5 % solvent and were added to the potato dextrose broth (PDB) medium to final concentrations of 31.25, 62.5, 125, 250, 500, 1.000, 2.000, 4.000 µg/mL, respectively. A 10 µl spore suspension (1.0 x 10^8^ spores/ mL) of each test pathogens was inoculated into the test tubes in PDB medium and incubated at 25 °C for 2-7 days. The control tubes containing PDB medium were inoculated only with fungal spore suspension. The standard reference fungicide, benomyl, was used as the positive control for the plant pathogens tested. The minimum concentrations at which no visible growth was observed were defined as the MICs and were expressed in µg/mL.

#### Spore germination and growth kinetics assay 

For spore germination assays of *B. cinerea, F. oxysporum, V. dahliae, A. oryzea *and* F. solani*, test samples of the leaf essential oil (4 µL) were dissolved in 5% DMSO to obtain 31.25, 62.5, 125, 250, 500, 1,000 and 2,000 µg/mL concentrations of the oil, where the final concentration of the solvent was 0.5 %. The samples were inoculated with the spore suspension of each fungal pathogen containing 1.0 x 10^8^ spores/mL. From this, aliquots of 10 µL spore suspension from each were placed on separate glass slides in triplicate. Slides containing the spores were incubated in a moisture chamber at 25 ± 2 °C for 4 h. Each slide was then fixed in lactophenol cotton blue and observed under the microscope for spore germination. The spores that had generated germ tubes were counted and the percentage of spore germination was calculated. The control dimethylsulfoxide (0.5 %) was tested**.**

*F. oxysporum*, which appeared to be a more resistant fungus than *B. cinerea* and *F. solani* to the leaf essential oil in spore germination study, was chosen as the test fungus for the kinetic study and evaluation of antifungal activity of the leaf essential oil (Rana et al., 1997[22]). A 10 µL spore suspension (1.0 x 10^8^ spores /mL) of this fungal pathogen was inoculated into different concentrations of the leaf essential oil (31.25, 62.5, 125 and 250 µg/mL) in a test tube and a homogenous suspension was made by inverting the test tubes 3-4 times. After the specific intervals 0, 30, 60, 90, 120 and 150 min, the reaction mixture was filtered through Whatman No. 1 filter paper and the retained spores were washed two or three times with sterile distilled water. The filter was then removed and spores were washed off into 10 mL of sterile distilled water. From this, 100 µl of the spore suspension was placed on a glass slide and incubated at 25 °C for 24 h. The spores that had generated germ tubes were counted and percentage of spores that had germinated was calculated. Control sets were prepared in 0.5 % DMSO with sterile distilled water. All experiments were conducted in triplicate.

#### In vivo antifungal activity assay

Further, to confirm the potential efficacy of the essential oil of *R. montana *leaf, the tomato plants (*Lycopersicon esculuntum*) were used as the host plants for the *in vivo *study. *B. cinerea *was selected as the test fungus which causes symptoms of grey mould of tomato plants. 

In brief, for the *in vivo* study, tomato plants were kept under ambient conditions. Further, to prepare the test solutions at a concentration of 1,000 µg/mL, 4 µl of essential oil was dissolved in 5 % dimethylsulfoxide (DMSO) followed by diluting it with water containing the surfactant Tween 20 (200 µg/mL), where the final concentrations of DMSO and Tween 20 were 0.5 and 0.1 %, respectively. The initial concentration of the test solution was 1.000 µg/mL, in further; test dilutions of 500 and 250 µg/mL of essential oil were employed. For applying the test samples of the oil, 4 mL of each test sample solution was sprayed into each pot at the same time. Further, 6 mL of fungal spore suspension (1.0 x 10^8^ spores/ mL ) of *B. cinerea *was sprayed onto each pot. Controls were sprayed with DMSO (0.5 %) and Tween 20 (0.1 %) solutions. Benomyl was used as a reference positive control. The area of lesions on treated plants was measured in millimetre using a Vernier calliper. All tests were conducted in three replicates. The effect of antifungal efficacy of the test samples on the disease was evaluated after 12 days as a percentage of inhibition calculated by the formula: 

Percent inhibition (%) = ((A-B)/A) x 100

where A and B represent the disease area on the 18 untreated and treated plants, respectively.

#### In vivo antibacterial assay

For this experiment, we use a highly infected soil by *A. tumefaciens *C58. The soil was maintained at 60 % of its water-holding capacity, and placed in plastic pots (30x20x15 cm, 2 kg of soil per pot) after adding 4 mL essential oil (1000, 500 or 250 µg/mL). Subsequently, 30 post-emerging bitter almond plants after root running were transplanted. Pots were monitored for a period of 3 months after transplantation under ambient conditions. Galls were inspected and weighted.

#### Microbial estimation

Ten grams of the soil sample were suspended in an Erlenmeyer flask containing 90mL of a sterile solution (0.2 % sodium polyphosphate (Na PO_3_)n in distilled water, pH 7). The flask was shaken at 250 rpm for 2 h. Serial ten fold dilutions of the samples in 0.9 % NaCl solution were plated in triplicate on LB medium for *Agrobacterium* and total bacterial counts respectively. Each soil sample was analysed twice and the dilution series were plated in triplicate for each experiment. All these counts were expressed as colony-forming units (CFU)/g of dried soil. Individual colonies on LB medium were re-streaked on LB, verified for purity and identified using the Gram coloration, API test (BioMerieux SA, France).

### Gas chromatography-mass spectrometry (GC-MS) analysis

The GC-MS analysis of the essential oil was performed using a SHIMADZU GC-MS (GC-17A) equipped with a ZB-1 MS fused silica capillary column (30 m 0.25 m i.d., film thickness 0.25 µm). For GC-MS detection an electron ionization system with ionization energy of 70 eV was used. Helium gas was used as the carrier gas at a constant flow rate of 1 mL/min. Injector and MS transfer line temperature were set at 220°C and 290°C, respectively. The oven temperature was programmed from 50 °C to 150 °C at 3 °C/min, then held isothermal for 10 min and finally raised to 250 °C at 10 °C/min. Diluted samples (1/100, v/v, in methanol) of 1.0 µl were injected manually in the splitless mode. The relative percentage of the oil constituents was expressed as percentages by peak area normalization.

Identification of components of the essential oil was assigned by comparison of their retention indices, relative to a series of n-alkane indices on the ZB-1 capillary column and GC-MS spectra from the Wiley 6.0 MS data and literature data and whenever possible, by co-injection with authentic compounds (Joulain and Konig, 1998[16]; Abdellatif et al., 2014[1]).

### Statistical analysis

Percentage data were transformed to arcsine values and the analysis of variance was carried out (ANOVA). The data were statistically analyzed and mean values were calculated. Analysis of variance for individual parameters was performed using Duncan's multiple range test, on the basis of mean values to find out the significance at p 0.05.

## Results

### Antifungal activity

The leaf essential oil of *R. montana* exhibited a moderate to high antifungal activity against all the plant pathogenic fungi tested. These findings prove that the leaf essential oil (1,000 µg/disk) showed a potent inhibitory effect against the growth of *F. oxysporum* (69 %), *B. cinerea* (80 %), *A. oryzea*, (40 %), *V. dahliae*, (60 %), *R. solani* (58 %) and *F. solani* (76 %). The growth of *B. cineria*, *F. solani* and *F. oxysporum* was significantly inhibited by the leaf essential of *R. montana* as compared to other plant pathogenic fungi tested. On the other hand, the leaf extract in methanol (1.500 µg/disk) exhibited significantly better antifungal effect as a percentage of the radial growth inhibition against all the plant pathogens tested as compared to ethyl acetate, chloroform and hexane extracts, ranging from 40 to 58%. Chloroform and ethyl acetate extracts also exerted a potential antifungal effect against all the plant pathogens tested with their respective radial growth inhibition percentages of 20-42.1 % and 18-53 %. However, the hexane extract did not reveal significant results of antifungal activity. A low to moderate antifungal effect of hexane extract was observed against some of the plant pathogens with radial growth inhibition percentage of 12.4-18.3 %. All the extracts showed less susceptibility against A. oryzea with a radial growth inhibition percentage of 5-15 %.

### Antifungal susceptibility

**Figure 1:**
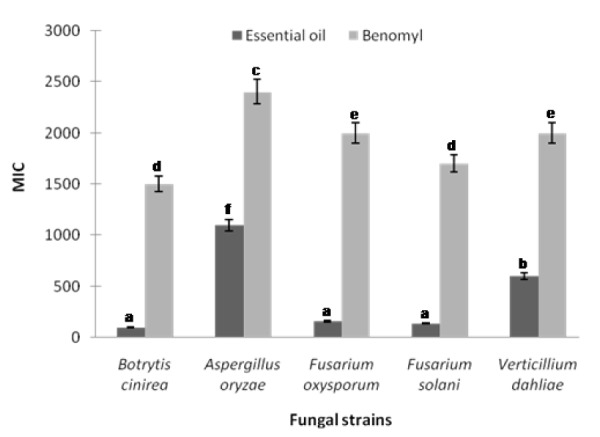
Minimum inhibitory concentration of the leaf essential oil of *R. montana* against plant pathogenic fungi. Each experiment was performed three times and the data averaged (n = 3). Numbers followed by different letters (a-f) are significantly different at the level of P<0.05 according to the Duncan’s multiple range tests. Benomyl was used as positive control for each pathogen.

**Figure 2:**
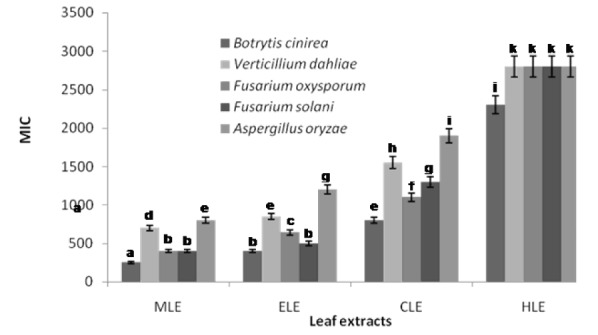
Minimum inhibitory concentration of the leaf extracts of *R. montana* against plant pathogenic fungi. MLE methanol leaf extract, ELE ethyl acetate leaf extract, CLE chloroform leaf extract, HLE hexane leaf extract. Each experiment was performed three times and the data averaged (n = 3). Numbers followed by different letters (a–k) are significantly different at the level of P < 0.05 according to the Duncan’s multiple range tests.

The minimum inhibitory concentrations (MICs) defined as the lowest concentrations of the leaf essential oil that results in complete growth inhibition of F. oxysporum, F. solani, B. cinerea, V. dahliae, and A. oryzea were found to be 160, 140, 100, 600 and 1100 µg/mL, respectively (Figure 1(Fig. 1)). *B. cinerea* was found to be the fungal pathogen most susceptible to the leaf essential oil of *R. montana*. In most of the cases in this study, the leaf essential oil exhibited a significantly higher antifungal effect than that of standard benomyl in regard to the plant pathogenic fungi tested (Figure 1(Fig. 1)). Also the leaf extracts of methanol, ethyl acetate and chloroform displayed potential antifungal effect at minimum inhibitory concentrations against all the plant pathogens tested. The MIC values of methanol, ethyl acetate and chloroform extracts ranged from 250 to 800, 400 to 1200 and 800 to 1900 µg/ mL, respectively (Figure 2(Fig. 2)). As a control, the solvent did not effect the growth of the fungal pathogens at the concentration used in this study. However, hexane extract did not show any potential effect of antifungal activity as a minimum inhibitory concentration.

### Spore germination

The results obtained for the leaf essential oil of *R. montana *from the spore germination assay of each of the test fungi revealed that Dimethylsulfoxide (DMSO) (0.5 %, v/v) as a control did not inhibit the spore germination of any of the plant pathogens tested. There was a significant inhibition of fungal spore germination at varied concentrations of the leaf essential oil. The oil showed significant antifungal effect as fungal spore germination inhibition and 100 % inhibition of fungal spore germination was observed against *F. oxysporum, F. solani, B. cinerea* at 1.000, 500 and 250 µg/mL concentrations of the leaf essential oil, respectively as compared to other plant pathogens tested. However, the oil also exhibited a potent inhibitory effect on the spore germination of* V. dahlia *and* A. oryzea *within the range of 30-55 % at concentrations ranging from 250 to 1.000 µg/mL. In addition, the results showed that the spore germination of all the tested pathogens depended significantly on the concentration of the oil (Table 1(Tab. 1)).

### Antifungal growth kinetics

Exposure of *F. oxysporum* spores to different concentrations of the oil for a period of 0-150 min caused varying degrees of inhibition of spore germination. An increase in fungicidal activity was observed with an increase in exposure time and concentration.

**Figure 3:**
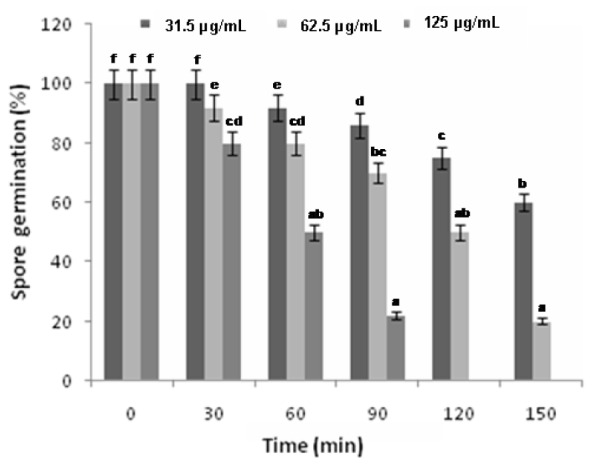
Kinetics of inhibition of *F. oxysporum* spores by the different concentrations of the leaf essential oil of *R. montana*. Each experiment was performed three times and the data averaged (n = 3). Numbers followed by different letters (a–f) are significantly different at the level of P < 0.05 according to the Duncan’s multiple range test.

The leaf oil at 31.25 µg/mL showed antifungal activity but not a rapid killing of the fungi and about 25 % inhibition was observed at an exposure time of 120 min. However, there was a significant increase in the killing rate at 62.5 and 125 µg/mL after 30 min of exposure. A significant correlation effect was observed between the time of exposition and the concentrations of the oil against* F. oxysporum* spores. 80 % and 100 % inhibition of spore germination using the leaf oil at 62.5 and 125 µg/mL respectively were observed after 150 min exposure (Figure 3(Fig. 3)**)**. The linear correlation coefficient values (r^2^) of the effect of varied concentrations of the oil and time against *F. oxysporum* spores were noted to be 0.9333, 0.9369, and 0.9535 which were closer to 1.0, confirming a good relationship between time and spore germination.

### In vivo antifungal activity

*In vivo* antifungal activity of the essential oil of *R. montana *against *B. cinerea* was assessed by the presence or absence of disease area on the tested tomato plants. According to the results given in Table 1(Tab. 1), the oil exhibited wide range of antifungal activity. The blind controls DMSO (0.5 %) and Tween 20 (0.1%) did not inhibit the growth of the test strain. At the initial concentration of 1.000 µg/mL the oil exhibited 100 % antifungal effect against leaf spot/scorch of tomato caused by *B. cinerea *as compared to positive control benomyl. Further concentrations of the oil applied to the plants were 500 and 250 µg/mL. Also at the concentration of 500 µg/mL, potential antifungal effect of the oil was observed with a 100 % antifungal effect against *B. cinerea*. However, the oil at the concentration of 250 µg/mL had a moderate antifungal effect (87.39 %) against *B. cinerea* on grown tomato plants (Table 2(Tab. 2)). It was observed that the antifungal effect of leaf essential oil was rapid and exhibited a significantly higher antifungal effect than the reference standard benomyl (Table 2(Tab. 2)).

### Suppression of crown gall disease by essential oil in pot experiments

Plants of bitter almond potted in a highly infected soil treated with different concentration of essential oil did not show symptoms of crown gall, while plants transplanted in untreated soil showed galls both on stems and roots. The weight of collected galls was approximately 18 mg/plant. The enumeration of the *A. tumefaciens* population in the treated soil by the addition of essential oil was significantly reduced (**< **10 CFU/g of soil). 

### Chemical composition of essential oil

GC-MS analyses of the oil led to the identification of 23 different components, representing 87.13 % of the total oil. The identified compounds are listed in Table 3(Tab. 3) according to their elution order on a ZB-1 capillary column. The oil contained a complex mixture consisting of olefinic hydrocarbons, mono- and sesquiterpenes and mono- and sesquiterpene hydrocarbon along with some other essential phytochemicals. The major components in the oil detected were 1-butene (38.33 %), methylcyclopropane (15.47 %), 2-butene (22.56 %) and caryophyllene oxide (8.18 %). Terpenoid hydrocarbons were the characteristic constituents of the oil of *R. montana*. Coumarin (0.18 %), eugenol (0.13 %), α-humulene (0.05 %), farnesol (0.02 %), linalool (0.08 %), benzene acetic acid (0.57 %), isovaleric acid (0.05 %), 2-butanone (0.07 %) and acetophenone (0.08 %) were also found to be the trace or minor components of *R. montana* oil in the present study.

## Discussion

In this study, the essential oil of *R. montana *obtained by hydrodistillation of the leaves contains monoterpene hydrocarbons and oxygen containing mono- and sesquiterpenes. In recent years, several researchers have reported the mono- and sesquiterpenes as the major components of various essential oils of plant origin, which have enormous potential for strongly inhibiting the growth of microbial pathogens (Gudzic et al., 2002[14]; Cakir et al., 2004[8]). In general, the active antimicrobial compounds of essential oils are terpenes, which are phenolic in nature, it would seem reasonable that their antimicrobial or antifungal mode of action might be related to that of other compounds. Most of the studies on the mechanism of phenolic compounds have focused on their effects on cellular membranes. Actually, phenolic compounds not only attack cell walls and cell membranes, thereby affecting the permeability and release of intracellular constituents but they also interfere with membrane function. Thus, active phenolic terpenes might have several invasive targets which could lead to the inhibition of plant pathogenic fungi. 

This research work also describes the complex effect of the oil on fungal spore germination and exhibited a wide range of antifungal activity. During the kinetic study of *F. oxysporum*, it appeared that exposure time of the oil had a little effect on the fungicidal activity at lower concentration but at a concentration of 125 µg/mL, the fungicidal action was very rapid and showed 100% spore germination inhibition of *F. oxysporum*. However, one of the fungal pathogens *A. oryzea* displayed less susceptibility to the oil in the present study. This might be ascribed to the existence of a high percentage of monoterpenes and monoterpene hydrocarbons reflecting that there was no significant correlation between the activity and the percentage of some major components identified (Cakir et al., 2004[8]). Also the results of the antifungal screening showed that leaf extracts of methanol, ethyl acetate and chloroform have strong antifungal activity against the tested plant pathogens. However, the hexane extract did not reveal significant results of antifungal activity. This might be attributable to the resistant behaviour of the test fungi against a low-polar extract. On the other hand, in the present study, the essential oil of *R. montana *showed potential *in vivo* antifungal effects against the tested plant pathogen of* B. cinerea* on tomato plants. Earlier *in vivo* studies on the analysis of antifungal effect of various oil/extracts showed that they had varying degrees of antifungal effect against different plant pathogenic fungi (Bajpai et al., 2009[3]; Yoo et al., 1998[30]). Previous research literature on the analysis and antifungal properties of the essential oils of various species have shown that they have varying degrees of growth inhibitory effects against some Fusarium, Botrytis and Rhizoctonia species due to their different chemical compositions (Alvarez-Castellanos et al., 2001[2]; Bouchra et al., 2003[6]). The oil of seven Moroccan Labiatae, which consists mainly of carvacrol, linalyl acetate, and thymol as major components, exhibited a complete mycelial inhibition effect on the growth of *B. cinerea* (Bouchra et al., 2003[6]). However, the oil of *Curcuma longa*, which consists mainly of sesquiterpenes and whose major constituents were α-turmerol, β-bisabolene, and β-caryophyllene, exhibited a complete mycelial growth inhibition against *F. oxysporum* (Singh et al., 2002[25]). Likewise, lavender and rosemary essential oils were fungitoxic to *F. solani*, although they had a different chemical composition (Alvarez-Castellanos et al., 2001[2]). In the essential oils of *Silene armeria L.,* 1-Butene, 2-Butene, Methylcyclopropane and Caryophyllene oxide were found as major components and some of them were also characterized in terms of the high contents of *R. montana* essential oil in the present investigation. The significant reduction of crown gall incidence on bitter almond rootstocks could be attributed to the presence those components present in the oil.

Certain oils and plant extracts including phytochemicals act in many ways on various types of disease complex, and may be applied in food and agricultural industries in the same way as other chemical fungicides. Mediated oil and extracts from *R. montana* leaves can also be used as a leading factor in a wide range of activities against many plant pathogenic fungi, where these pathogens have developed resistance against specific fungicides (benzimidazoles, dicarboximides, diethofuncarband and the sterol biosynthesis inhibitors) (Elad et al., 1991[13]). In addition, it is also possible that the trace/minor components, such as coumarin, eugenol, α-humulene, farnesol, linalool, pentylfuran, benzene acetic acid, 2-butanone and acetophenone might be involved in some type of antifungal synergism with other active components of essential oil and these findings are in agreement with previous reports (Marino et al., 2001[17]; Hutchings et al., 1996[15]). Thus, it can be concluded that the use of *R. montana* mediated oil and extracts could be considered as an antimicrobial agent available for developing a novel type of natural fungicide and bactericide able to control several plant pathogenic fungi and bacteria causing severe diseases in food, crops and vegetables.

## Acknowledgements

This work was supported by the Ministry of Agriculture and Water Resources and the Ministry of Higher Education and Scientific Research, Tunisia. 

## Conflict of interest

The authors report no conflict of interest. The authors are responsible for the content and writing of the paper.

## Figures and Tables

**Table 1:**
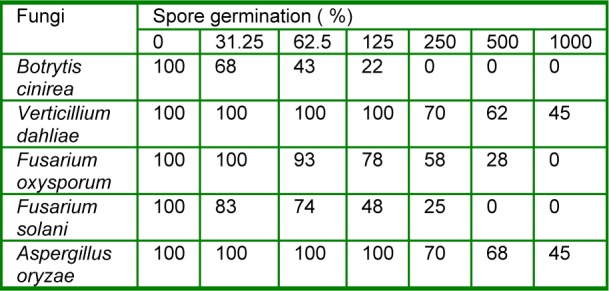
Effect of different concentrations (µg/mL) of the leaf essential oil of *R. montana* on spore germination of tested fungi.

**Table 2:**
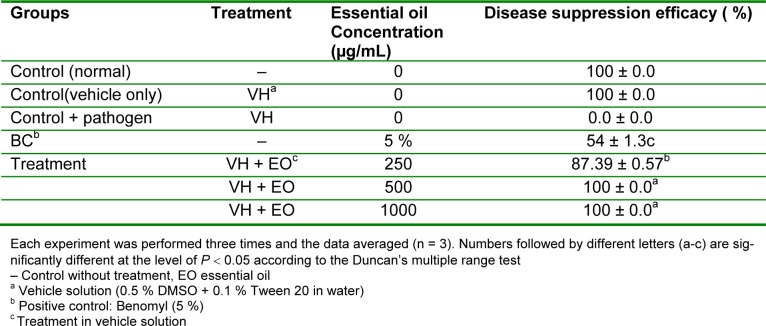
*In vivo* antifungal activity of leaf essential oil of* R. montana* against the plant pathogenic fungi *B. cinerea* on tomato plants

**Table 3:**
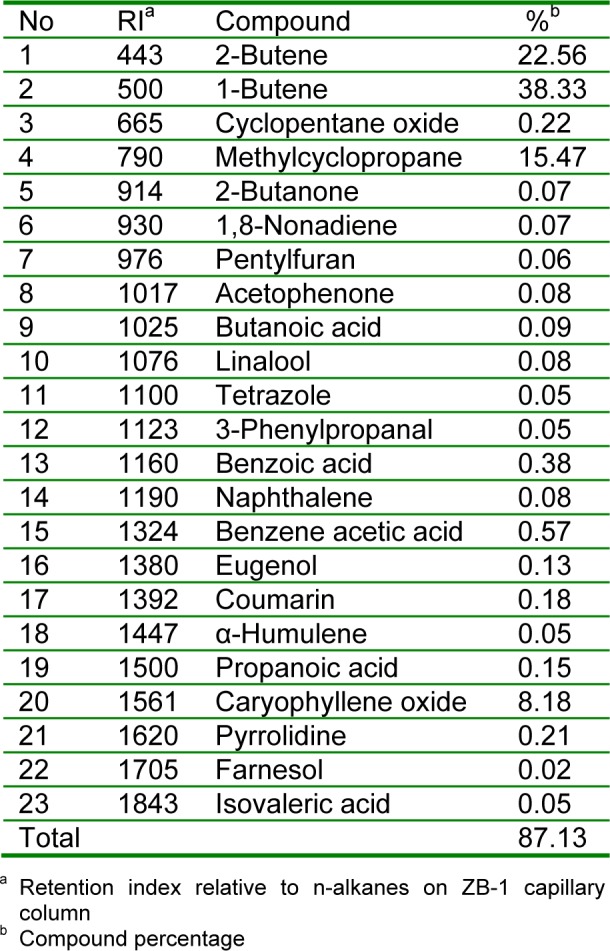
Chemical composition of volatile oil isolated by hydrodistillation from *R. montana*
